# Therapeutic and chemopreventive potential of melatonin in liver diseases: targeting oxidative stress and inflammatory signaling

**DOI:** 10.3389/fphar.2026.1931181

**Published:** 2026-09-10

**Authors:** Md Ataur Rahman, Deokyong Sim, Sujay Kumar Bhajan, Anup Kumar Bishwas, Maroua Jalouli, Jinwon Choi, Nahida Aktary, Sohyun Park, Ilsun Yoo, Amama Rani, Abdel Halim Harrath, Sung-Hoon Kim, Bonglee Kim

**Affiliations:** 1 Department of Oncology, Karmanos Cancer Institute, Wayne State University, Detroit, MI, United States; 2 Department of Pathology, College of Korean Medicine, Kyung Hee University, Seoul, Republic of Korea; 3 Department of Biotechnology and Genetic Engineering, Faculty of Life Sciences, Gopalganj Science & Technology University, Gopalganj, Bangladesh; 4 Department of Biology, College of Science, Imam Mohammad Ibn Saud Islamic University (IMSIU), Riyadh, Saudi Arabia; 5 Department of Zoology, College of Science, King Saud University, Riyadh, Saudi Arabia; 6 Dr. B Lab Co., Ltd. Samuiwon Startup Center Kyung Hee University, Seoul, Republic of Korea

**Keywords:** antioxidant signaling, chemopreventive, fibrosis, hepatocellular carcinoma, liver diseases, melatonin, Nrf2

## Abstract

The hormone melatonin, secreted by the pineal gland, has attracted considerable interest due to its potential to prevent liver disorders by its strong antioxidant characteristics. This review examines the diverse functions of melatonin in maintaining liver health, with a specific emphasis on its mechanisms of action in different liver diseases. Melatonin counteracts the harmful effects of alcohol on the liver by reducing oxidative stress and maintaining the proper functioning of mitochondria. Melatonin improves insulin sensitivity and regulates lipid metabolism in metabolic liver diseases, such as non-alcoholic fatty liver disease (NAFLD), and decreasing hepatic steatosis. The anti-inflammatory properties of this substance are obtained by reducing pro-inflammatory cytokines signaling pathways, which protect against diseases such as hepatitis and steatohepatitis. In addition, melatonin demonstrates anti-fibrotic characteristics by inhibiting the activation of hepatic stellate cells and decreasing the production of fibrogenic cytokines, reducing the severity of liver fibrosis. Within the realm of hepatocellular carcinoma (HCC), melatonin triggers programmed cell death that hinders the growth of cells and represses the formation of new blood vessels, augmenting the effectiveness of traditional treatments. Research has shown that melatonin is safe and effective in treating liver problems. However, further large-scale, multicenter randomized clinical trials are required to establish the optimal therapeutic dose, treatment duration, long-term safety, and clinical efficacy of melatonin in patients with liver diseases. Future research should focus on clarifying the specific molecular targets of melatonin, enhancing its bioavailability, and creating combination therapies. The existing evidence indicates that melatonin is a potentially beneficial supplementary treatment and preventive medication for liver disorders, owing to its antioxidant, anti-inflammatory, anti-fibrotic, and metabolic regulating characteristics. Nonetheless, additional well designed clinical trials are necessary to determine its ideal therapeutic use and promote its integration into clinical practice.

## 1 Introduction

Liver diseases pose a substantial worldwide health issue, leading to significant levels of infection and death (Manikat et al., 2024; Paulino et al., 2024). These disorders include a wide range of conditions, such as alcoholic liver disease (ALD), non-alcoholic fatty liver disease (NAFLD), hepatitis, liver fibrosis, and hepatocellular carcinoma (HCC) (Vaz et al., 2024; Ye et al., 2024). Liver diseases typically advance due to intricate oxidative stress, inflammation, and fibrosis interplays. Existing therapy approaches generally concentrate on symptom management and the deceleration of disease advancement. However, there is a pressing demand for potent chemopreventive medicines that can specifically address the underlying pathogenic pathways (Singh et al., 2023).

Melatonin, a hormone predominantly secreted by the pineal gland, has garnered interest due to its potential in preventing and treating liver disease (Hussain et al., 2024; Hong and Kim, 2024). Melatonin, formally called Methoxy Tryptamine, is mainly known for controlling circadian rhythms and sleep-wake cycles (Arnao et al., 2023; Jumnake et al., 2024). Nevertheless, a thorough investigation conducted in recent decades has unveiled its diverse biological functions, encompassing potent antioxidant, anti-inflammatory, and anti-fibrotic characteristics. These properties render melatonin a promising choice for alleviating the pathophysiology of liver disease.

The liver is very vulnerable to oxidative stress because of its crucial function in detoxifying and metabolic processes (Rauf et al., 2024; Banerjee et al., 2023), which produce reactive oxygen species (ROS). An excessive amount of ROS can cause harm to the various components within cells, resulting in injury and death of liver cells. The antioxidant effects of melatonin are mainly achieved by directly scavenging free radicals and increasing the production of antioxidant enzymes (Boutin et al., 2024; Zhao and Hu, 2023). This simultaneous process aids in preserving the equilibrium of cellular redox, safeguarding hepatocytes from oxidative damage. Melatonin not only has antioxidant properties but also demonstrates substantial anti-inflammatory activity (Frungieri et al., 2024). Chronic inflammation is a characteristic feature of numerous liver illnesses, playing a role in causing tissue damage and fibrosis. Melatonin modulates inflammatory responses by inhibiting the production of pro-inflammatory cytokines and suppressing the activation of key inflammatory signaling pathways (Ozturk et al., 2023; Hosseinzadeh et al., 2024), such as nuclear factor kappa B (NF-κB). Melatonin can suppress inflammation, which can halt the advancement of liver damage and enhance the general functioning of the liver (Won et al., 2021).

Subsequent research on melatonin in hepatic disorders should concentrate on clarifying its specific molecular mechanisms, targeting biomarkers for patient classification, and creating novel melatonin analogs with better bioavailability. Comprehending the synergistic effects of melatonin alongside other medicinal drugs may facilitate the development of more efficacious methods for treatment (Yu et al., 2021a; Wang et al., 2022). Moreover, exploring the prospective benefits of melatonin in the treatment of additional liver conditions, including viral hepatitis and autoimmune liver diseases, may enhance its therapeutic applicability (Juybari et al., 2020). This review examines the characteristics of melatonin, which demonstrates significant potential as a chemopreventive and therapeutic agent for liver disorders. Its antioxidant, anti-inflammatory, anti-fibrotic, and anti-cancer properties offer a holistic strategy for liver protection and the management of associated disorders. Consequently, additional research and thorough testing will be crucial for translating these findings into actual medical applications, thereby improving the lives of patients and overall wellbeing.

## 2 Melatonin synthesis, metabolism, and biological functions

**FIGURE 1:**
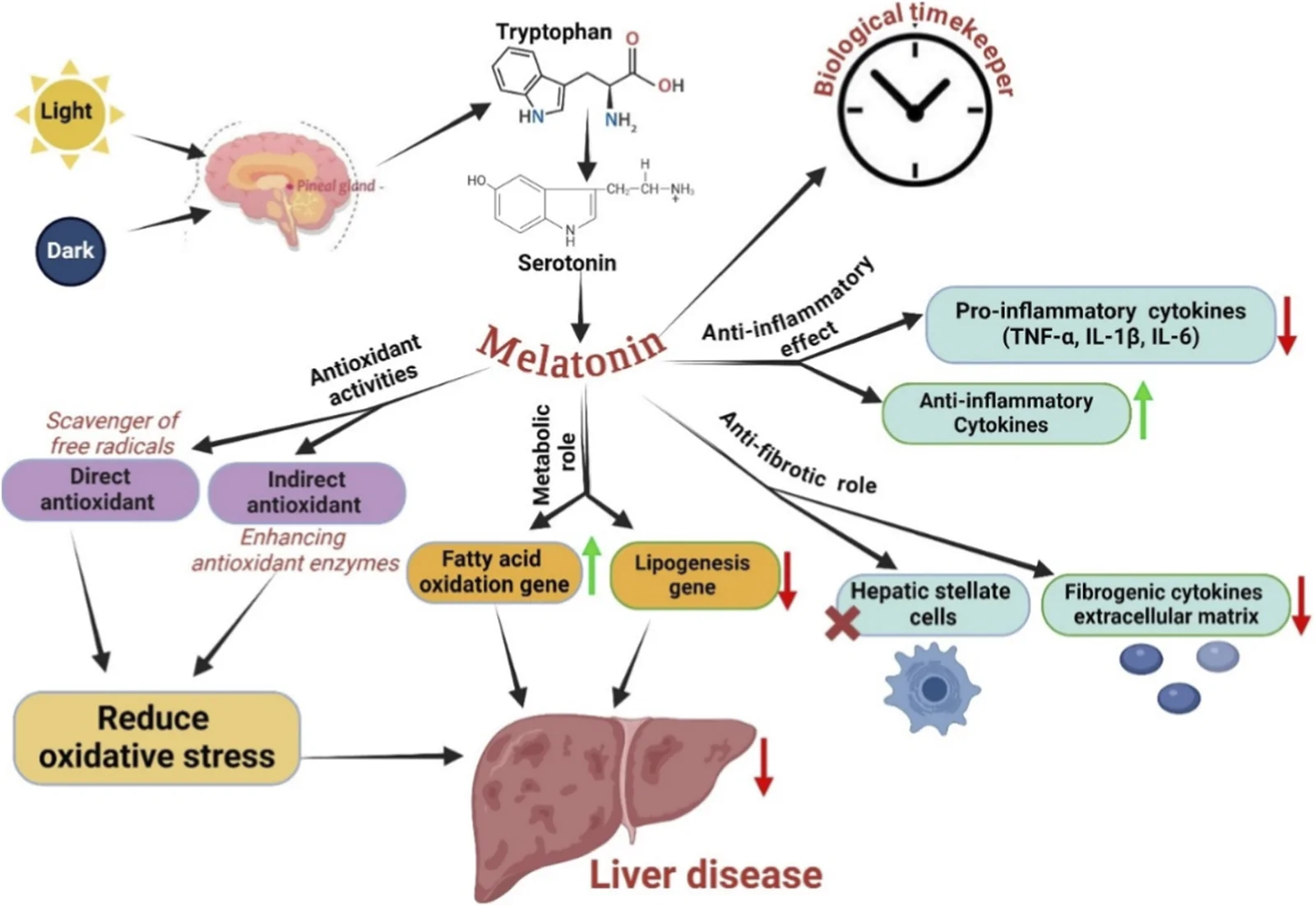
Overview of melatonin synthesis pathway and their diverse biological functions. The pineal gland in the brain secretes Melatonin via a series of reactions, which largely depend on light and darkness. Melatonin mainly performs four distinct biological functions: antioxidant, anti-inflammatory, metabolic, and anti-fibrotic. Antioxidant properties: Melatonin is a direct scavenger of free radicals and enhances the activity of other antioxidant enzymes (superoxide dismutase, glutathione peroxidase, and catalase) that reduce oxidative stress and liver disease. Also, Melatonin decreases pro-inflammatory cytokines and increases anti-inflammatory cytokines that prevent liver disease. In addition, Melatonin plays a pivotal role by regulating the expression of fatty acid oxidation genes and downregulating the lipogenesis genes. Moreover, Melatonin hinders the generation of hepatic stellate cells and decreases fibrogenic cytokines and extracellular matrix components, preventing and improving liver fibrosis.

Melatonin, often referred to as N-acetyl-5-methoxytryptamine, is primarily synthesized by the pineal gland located in the brain (Murala et al., 2022). The synthesis and secretion of this substance are rigorously controlled by the light-dark cycle, with its production reaching its highest point during the night (Woldańska-Okońska and Koszela, 2024). The fundamental function of melatonin is to regulate circadian rhythms, specifically the sleep-wake cycle, which is essential for sustaining both physiological and psychological wellbeing (Rutkowska et al., 2024). The production of melatonin initiates with the amino acid tryptophan, which undergoes a sequence of enzymatic transformations to serotonin and ultimately to melatonin (Zauq et al., 2024; Kwon et al., 2024). The process is susceptible to changes in environmental light since it is suppressed by exposure to sunlight and enhanced in darkness. This distinctive regulatory system supports melatonin’s function as a biological timekeeper, synchronizing the body’s internal clock with the external surroundings (Liu and Xuan, 2024). Recent findings have revealed that the pineal gland is not the sole producer of melatonin. Substantial amounts of melatonin are produced in extra-pineal tissues, including inside the gastrointestinal system and mitochondria, where it executes crucial local physiological roles without reliance on circadian control (Cao et al., 2025). The gastrointestinal system harbors melatonin levels that significantly exceed those found in the pineal gland and bloodstream, with enterochromaffin cells being the principal site of production (Subakathulla et al., 2026). In contrast to pineal melatonin, gastrointestinal melatonin is primarily influenced by dietary consumption rather than the circadian rhythm, functioning through autocrine and paracrine pathways to uphold intestinal barrier integrity, modulate immunological responses, and sustain gut microbial equilibrium (Gonciarz et al., 2026). These measures are crucial for preserving the gut-liver connection, thereby reducing endotoxin migration, liver inflammation, and metabolic impairment. Furthermore, contemporary findings suggest that mitochondria serve as both a primary target and an intracellular origin of melatonin (Bao et al., 2026). Mitochondrial melatonin directly neutralizes reactive oxygen and nitrogen species produced during oxidative phosphorylation, maintains mitochondrial membrane potential, safeguards mitochondrial DNA from oxidative damage, boosts ATP production, and modulates mitophagy and apoptosis (Rahman et al., 2025). The functions particular to mitochondria are significantly pertinent to liver physiology, as hepatocytes possess a high density of mitochondria and are perpetually subjected to oxidative stress resulting from xenobiotic metabolism and fatty acid oxidation (Rahman et al., 2024). Thus, extra-pineal and mitochondrial melatonin offer a new molecular foundation for the robust antioxidant, anti-inflammatory, anti-fibrotic, and metabolic regulatory functions of melatonin noted in both experimental and clinical investigations of hepatic disorders (Dong et al., 2025). Melatonin biosynthesis in the pineal gland and extra-pineal tissues, including the gastrointestinal tract and mitochondria, hepatic metabolism and pharmacokinetics of melatonin, and receptor-dependent and receptor-independent biological functions, including antioxidant, anti-inflammatory, metabolic, and anti-fibrotic mechanisms relevant to liver physiology. In addition to its well-established role in regulating sleep, melatonin has a diverse range of biological functions, principally due to its powerful antioxidant qualities. It functions as a direct scavenger of free radicals and an indirect antioxidant by enhancing the activity of other antioxidant enzymes (Ain et al., 2024; Reiter et al., 2024; Joseph et al., 2024), including superoxide dismutase, glutathione peroxidase, and catalase (Figure 1). These effects allow melatonin to reduce oxidative stress, which is crucial in developing various diseases, including liver ailments (de Almeida Chuffa et al., 2024).

Melatonin’s antioxidant capacity encompasses safeguarding cellular constituents, including lipids, proteins, and DNA, against oxidative harm (Obaid Saeed Alfalahi et al., 2024). The liver, a vital organ involved in metabolism and detoxification, is constantly subjected to oxidative stress. Therefore, this protective effect is especially crucial in safeguarding its functions (Conde de la Rosa et al., 2022). Mitochondrial function in the liver is upheld by melatonin, which prevents oxidative harm to mitochondrial DNA and proteins (Gunata et al., 2020; Zhang et al., 2024a). This action safeguards cellular energy generation and diminishes cell death. In addition, melatonin possesses significant anti-inflammatory capabilities. It regulates the immune response by suppressing the synthesis of pro-inflammatory cytokines, such as tumor necrosis factor-alpha (TNF-α) and interleukins (IL-1β, IL-6), and by increasing the production of anti-inflammatory cytokines, (Jiang et al., 2024; Gasmi et al., 2024; Vaghari-Tabari et al., 2023; Berbets et al., 2021). The immunomodulatory impact of this treatment aids in reducing chronic inflammation, a prevalent contributing factor in liver illnesses. Melatonin influences the expression of many genes engaged in metabolic pathways, therefore playing a pivotal role in liver metabolism (Samec et al., 2021). It upregulates the expression of genes related to fatty acid oxidation and downregulates the expression of genes involved in lipogenesis (Wang et al., 2021; Nath et al., 2024). These actions regulate lipid metabolism, crucial for avoiding and controlling diseases such as NAFLD. Melatonin possesses anti-fibrotic characteristics and antioxidant, anti-inflammatory, and metabolic regulating actions. It hinders the activation of hepatic stellate cells, which play a crucial role in the progression of liver fibrosis (Jia et al., 2024; Haddad Kashani et al., 2024). Melatonin aids in the prevention and improvement of liver fibrosis by decreasing the generation of fibrogenic cytokines and extracellular matrix components (Akhzari et al., 2022). Therefore, the various biological functions of melatonin highlight its potential as a treatment for liver problems. The capacity of this substance to control oxidative stress, inflammation, metabolism, and fibrosis makes it a desirable option for the prevention and treatment of many liver disorders. Thus, it justifies the need for additional study and clinical investigation.

### 2.1 Biosynthetic pathway, receptors, antioxidant mechanisms

Melatonin (N-acetyl-5-methoxytryptamine) is predominantly produced in the pineal gland via a well-preserved enzymatic route derived from the vital amino acid tryptophan (Li et al., 2025). Initially, tryptophan undergoes hydroxylation by tryptophan hydroxylase (TPH) to produce 5-hydroxytryptophan, which is then decarboxylated by aromatic L-amino acid decarboxylase (AADC) to yield serotonin (5-hydroxytryptamine) (Wang et al., 2023). Serotonin is subsequently acetylated by arylalkylamine N-acetyltransferase (AANAT), the enzyme that limits the rate of melatonin production, resulting in the formation of N-acetylserotonin (Kang et al., 2025). Ultimately, acetylserotonin O-methyltransferase (ASMT), alternatively referred to as hydroxyindole O-methyltransferase (HIOMT), facilitates the methylation of N-acetylserotonin to produce melatonin (Zhao et al., 2019). The function of AANAT is meticulously controlled by the circadian rhythm via sympathetic activation of the pineal gland, leading to elevated melatonin synthesis at night and diminished release during the day (Maitra and Pal, 2017).

Melatonin influences cellular processes via mechanisms that are both receptor-dependent and receptor-independent. The receptor-dependent process is predominantly facilitated by two G protein-coupled membrane receptors, MT1 (MTNR1A) and MT2 (MTNR1B), which are extensively found in liver tissue and immune cells (Nikolaev et al., 2021). The stimulation of these receptors modulates cyclic AMP signaling, protein kinase A, phospholipase C, and various downstream pathways associated with glucose metabolism, lipid balance, inflammation, apoptosis, and mitochondrial activity (Sethi et al., 2025). Nuclear receptors from the retinoid-related orphan receptor family (ROR/RZR) have been shown to play a role in certain genomic effects of melatonin, however their physiological function is still being explored (Romero, 2025).

A distinctive feature of melatonin is its antioxidant properties that do not rely on receptors. In contrast to traditional antioxidants, melatonin easily traverses cellular and mitochondrial membranes due to its amphiphilic characteristics (Bao et al., 2026). It actively neutralizes several reactive oxygen and nitrogen species, such as hydroxyl radicals, hydrogen peroxide, singlet oxygen, nitric oxide, and peroxynitrite (Reiter et al., 2002). Moreover, melatonin enhances intrinsic antioxidant defense mechanisms by engaging the Nrf2/Keap1 signaling pathway, resulting in elevated levels of antioxidant enzymes including superoxide dismutase (SOD), catalase (CAT), glutathione peroxidase (GPx), glutathione reductase, and heme oxygenase-1 (HO-1) (Silva et al., 2025). Concurrently, melatonin diminishes pro-oxidant enzymes and prevents oxidative harm to lipids, proteins, and DNA (Nuszkiewicz et al., 2026). The varied biological characteristics of melatonin establish it as a versatile signaling molecule that can safeguard the liver from oxidative stress, inflammation, metabolic disorders, fibrosis, and hepatocellular cancer.

### 2.2 Hepatic metabolism and metabolites

The liver is the principal organ accountable for melatonin metabolism, thus it is crucial in managing its systemic bioavailability and biological function. Upon release from the pineal gland or subsequent oral intake, melatonin experiences significant first-pass hepatic metabolism mostly via cytochrome P450 (CYP) enzymes (Zheng et al., 2025). CYP1A2 is the primary enzyme involved in the biotransformation of melatonin, whereas CYP1A1 and CYP2C19 play a minor role (Zheng et al., 2025). Melatonin is primarily transformed into 6-hydroxymelatonin, which is then conjugated by sulfotransferases to produce 6-sulfatoxymelatonin (aMT6s), the principal urine metabolite and a commonly utilized biomarker for endogenous melatonin secretion. A lesser fraction of melatonin is processed via kynuramine pathways, yielding biologically active metabolites such as N^1^-acetyl-N^2^-formyl-5-methoxykynuramine (AFMK) and N^1^-acetyl-5-methoxykynuramine (AMK), which possess significant antioxidant and anti-inflammatory properties (Simon et al., 2025). Hepatic impairment significantly modifies melatonin pharmacokinetics by diminishing metabolic clearance, extending its half-life, and elevating circulating melatonin levels, occurrences commonly seen in individuals with chronic liver disease and cirrhosis (Arjunan et al., 2022). These modifications could lead to impaired circadian rhythms, sleep irregularities, and metabolic dysfunctions linked to severe liver disease. Consequently, comprehending hepatic melatonin metabolism is crucial for refining therapeutic dose, reducing pharmacokinetic variability, and enhancing the hepatoprotective effectiveness of melatonin in individuals with liver ailments (Gonciarz et al., 2023).

## 3 Melatonin and alcohol in liver

Alcohol intake is a significant contributor to liver diseases, such as alcoholic fatty liver disease (AFLD), alcoholic hepatitis, and cirrhosis (Osna et al., 2024; Subramaniyan et al., 2021). These disorders arise due to long-term alcohol consumption, which triggers oxidative stress, lipid peroxidation, inflammation, and fibrosis in the liver. Melatonin has garnered interest due to its powerful antioxidant and anti-inflammatory characteristics. This makes it a possible contender for alleviating liver damage caused by alcohol consumption. Alcohol mainly causes damage to the liver by producing ROS and causing oxidative stress (Mandrekar and Mandal, 2024; Tan et al., 2020). The liver’s antioxidant defenses are overwhelmed by excessive formation of ROS, resulting in lipid peroxidation, mitochondrial malfunction, and cell damage. Due to its high lipophilicity and capacity to permeate cellular membranes, melatonin effectively removes ROS. It boosts the function of natural antioxidant enzymes like superoxide dismutase (SOD), glutathione peroxidase (GPx), and catalase (Chrustek and Olszewska-Słonina, 2021). The antioxidative effect of this action aids in maintaining the stability of cellular membranes, safeguarding the integrity of mitochondria, and decreasing lipid peroxidation. As a result, it helps maintain proper liver function. Table 1 elucidates the function of melatonin in many experimental and clinical scenarios, emphasizing its antioxidant and anti-inflammatory properties in alcohol-induced hepatic injury.

**TABLE 1:** Effects of melatonin on alcohol-induced liver damage: model, concentration, effectiveness, and mechanism.

Model	Doses	Efficacy	Mechanism	Ref
Alcoholic fatty liver disease (AFLD) - Animal models	10–20 mg/kg in animal studies	Reduces oxidative stress, lipid peroxidation, and inflammation	Melatonin reduces ROSformation, lipid peroxidation, and boosts natural antioxidant enzymes like SOD, GPx, and catalase	(Rabelo et al., 2024; Liu et al., 2017)
Alcoholic hepatitis - Animal models	10–50 mg/kg	Decreases pro-inflammatory cytokines, improves histology outcomes	Inhibits activation of nuclear factor-kappa B (NF-κB) and decreases levels of TNF-α and IL-6, reducing inflammation	(Scarlata et al., 2024; Kütük et al., 2024)
Alcohol-induced liver injury - Human clinical trials	3–5 mg/day orally (in clinical studies)	Improves liver function indicators, reduces oxidative stress markers	Enhances antioxidant defenses, decreases markers of oxidative stress, and reduces cytokine levels	(Izzo et al., 2021)
Gut-liver axis dysfunction - Animal models	10–20 mg/kg in animal studies	Reduces gut permeability, decreases endotoxin transfer, and minimizes liver inflammation	Strengthens intestinal barrier function, reducing endotoxin (LPS) translocation and subsequent liver inflammation	(Ge et al., 2024)

Furthermore, melatonin possesses antioxidant capabilities and plays a critical role in mitigating alcohol-induced liver impairment by exerting anti-inflammatory actions (LeFort et al., 2023). Chronic alcohol intake stimulates Kupffer cells, the liver’s resident macrophages, resulting in the synthesis and secretion of pro-inflammatory cytokines such as tumor necrosis factor-α (TNF-α), interleukin-1β (IL-1β), and interleukin-6 (IL-6) (Scarlata et al., 2024). These inflammatory agents exacerbate liver inflammation, facilitate hepatocyte damage, and play a role in the advancement of alcoholic liver disease. These cytokines sustain a continuous process of inflammation, which worsens liver damage. By inhibiting the activation of nuclear factor-kappa B (NF-κB), melatonin reduces the production of pro-inflammatory cytokines, molecules involved in inflammatory pathways (Chitimus et al., 2020; Lisboa et al., 2024). This control of the inflammatory response aids in reducing liver damage and enhancing overall liver wellbeing.

Moreover, the significance of melatonin in regulating the gut-liver axis is also noteworthy in the context of alcohol-induced liver disease (Ballway and Song, 2021). Alcohol impairs the function of the gut barrier, causing the intestines to become more permeable and allowing harmful substances like lipopolysaccharides (LPS) to move from the intestines into the liver. This mechanism initiates an inflammatory response and adds to the detriment of the liver. Studies have demonstrated that melatonin can improve the strength of the intestinal barrier and decrease the movement of endotoxins, which in turn helps to minimize liver inflammation (Moghadam Fard et al., 2024). Animal studies and clinical trials have shown evidence of the hepatoprotective properties of melatonin in alcohol-induced liver damage (Ullah et al., 2020). For example, when melatonin is given to animal models with alcoholic liver disease, it leads to a decrease in oxidative stress, a reduction in liver inflammation, and an improvement in histology outcomes. Human clinical trials have demonstrated that supplementing with melatonin can improve liver function indicators and decrease markers of oxidative stress and inflammation in individuals with alcoholic liver disease (Terziev and Terzieva, 2023). Overall, the antioxidant, anti-inflammatory, and gut-barrier-protecting qualities of melatonin make it a promising treatment option for alcohol-induced liver disorders. Further investigation should prioritize the optimization of dose regimens, the comprehension of long-term safety, and the incorporation of melatonin into comprehensive therapy plans for individuals with alcohol-related liver problems.

## 4 Melatonin and metabolism in the liver

**FIGURE 2:**
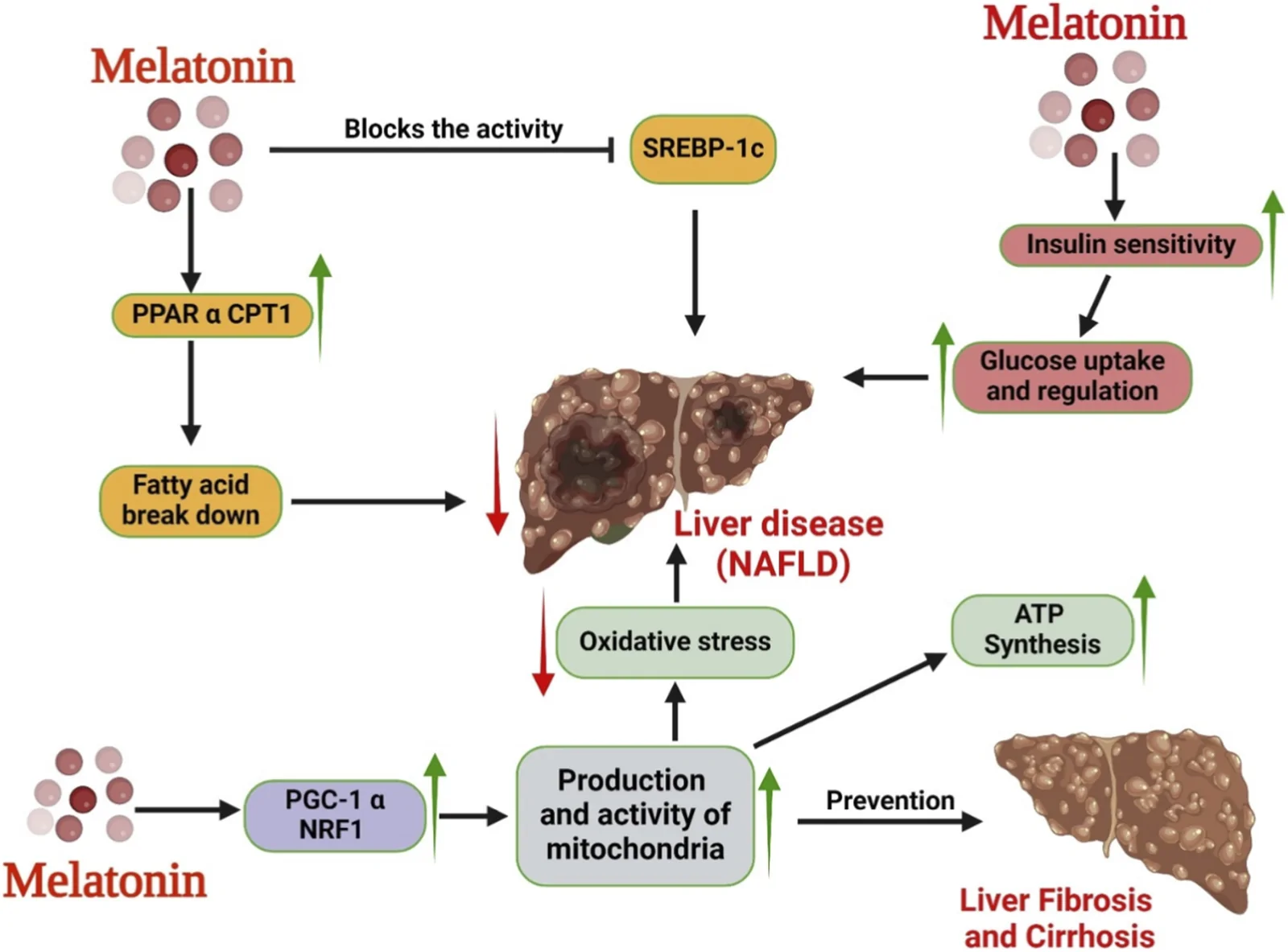
Metabolic function of melatonin in liver. The metabolic effects of melatonin on insulin sensitivity, mitochondrial function, and lipid metabolism demonstrate its potential as a therapeutic agent for treating liver illnesses like NAFLD. In NAFLD, insulin resistance, build-up of fat, and oxidative stress are common characteristics. Melatonin enhances the pathways responsible for insulin signaling, promoting glucose uptake and use. It also increases the activation of PPARa and CPT1 and halts fat production, consequently hindering the development of NAFLD. Moreover, NAFLD is halted by the increasing expression of PGC-1a and NRF1 regulators, which augments mitochondrial activity. This reduces oxidative stress, liver fibrosis, and cirrhosis and increases ATP synthesis.

Melatonin is essential for controlling liver metabolism, especially in diseases like NAFLD and metabolic syndrome (Gonciarz et al., 2023). NAFLD, the excessive buildup of fat in the liver, is strongly associated with insulin resistance, obesity, and dyslipidemia (Marušić et al., 2021; Muzurović et al., 2021). The impact of melatonin on liver metabolism is complex, encompassing the regulation of insulin sensitivity, lipid metabolism, and mitochondrial function. Melatonin improves liver metabolism by increasing insulin sensitivity (Obayemi et al., 2021). Insulin resistance is a crucial characteristic of metabolic diseases, including NAFLD, resulting in reduced ability of the liver to take up glucose and increased fat production. Studies have demonstrated that melatonin enhances the pathways responsible for insulin signaling, which in turn promotes the uptake and use of glucose (Tavares et al., 2021). This phenomenon aids in diminishing hepatic glucose synthesis and regulating systemic blood glucose levels, thereby alleviating one of the main catalysts of metabolic liver disorders. In addition, melatonin influences lipid metabolism by controlling the expression of genes responsible for lipid synthesis and degradation (Lei et al., 2024). It enhances the activation of peroxisome proliferator-activated receptor alpha (PPARα) and carnitine palmitoyltransferase 1 (CPT1), which play a vital role in the breakdown of fatty acids (Figure 2). At the same time, melatonin blocks the activity of sterol regulatory element-binding protein 1 (SREBP-1c), an essential protein that controls the production of fats in the body (Zhou et al., 2024). Melatonin decreases the buildup of triglycerides in the liver, relieving hepatic steatosis (Du et al., 2022). The following table provides an overview of the models, concentrations, effectiveness, and methods by which melatonin influences liver metabolism, namely, in disorders such as NAFLD and metabolic Syndrome (Table 2).

**TABLE 2:** Summarizing the effects of melatonin on liver metabolisms.

Model	Doses	Treatment duration	Efficacy	Mechanism	Ref
NAFLD (High-fat diet rat/mouse models)	5–20 mg/kg/day (oral or i.p.)	4–12 weeks	Improves insulin sensitivity, decreases hepatic glucose production and fasting glucose	Enhances insulin signaling (IRS-1/PI3K/Akt), improves glucose uptake, suppresses gluconeogenesis	(Pramanik et al., 2024)
NAFLD (High-fat diet models)	10–20 mg/kg/day	6–12 weeks	Reduces hepatic triglyceride accumulation and hepatic steatosis	Activates PPARα and CPT1, inhibits SREBP-1c, promotes β-oxidation	(Carvalho et al., 2024)
NAFLD/NASH (Animal models)	10 mg/kg/day	4–8 weeks	Improves mitochondrial function and ATP production, reduces oxidative stress	Upregulates PGC-1α, NRF1, enhances mitochondrial biogenesis, decreases mitochondrial ROS	(Chen et al., 2024)
Metabolic syndrome (Diet-induced obesity models)	10–30 mg/kg/day	6–12 weeks	Improves insulin resistance, lipid profile, and body weight	Activates AMPK, suppresses lipogenesis, improves energy metabolism	(Farias et al., 2019)
Hepatic oxidative stress (Experimental models)	10 mg/kg/day	2–8 weeks	Reduces lipid peroxidation and oxidative damage	Activates Nrf2/HO-1, increases SOD, CAT, GPx, decreases MDA	(Zhang et al., 2017)
Human clinical studies (NAFLD/metabolic disorders)	3–10 mg/day (oral)	8–24 weeks	Improves liver enzymes (ALT, AST), insulin resistance, and oxidative stress markers	Antioxidant, anti-inflammatory, improved mitochondrial function and lipid metabolism	(Yu et al., 2021b)

Mitochondrial activity, a crucial aspect of liver metabolism, is significantly influenced by melatonin. Mitochondria are key in energy production and metabolic balance, and their dysfunction is a characteristic of NAFLD. Melatonin boosts the production and activity of mitochondria by increasing the expression of important regulators such as PGC-1α and NRF1 (Faria et al., 2022). This improves mitochondrial respiration, increases ATP synthesis, and reduces oxidative stress. Maintaining healthy mitochondria is crucial in preventing the progression of NAFLD to more severe liver conditions like non-alcoholic steatohepatitis (NASH) and cirrhosis, and melatonin plays a vital role in this prevention (Ramanathan et al., 2022). The antioxidant properties of melatonin further support its metabolic benefits, particularly in preventing the progression of NAFLD.

Melatonin acts as a scavenger for free radicals and increases the production of antioxidant enzymes, decreasing oxidative harm to liver cells and enhancing liver function (Chrustek and Olszewska-Słonina, 2021; Martínez Soriano et al., 2020). To summarize, the regulatory effects of melatonin on insulin sensitivity, lipid metabolism, and mitochondrial activity demonstrate its promise as a therapeutic agent for treating metabolic liver disorders. The capacity of melatonin to regulate these crucial pathways emphasizes the significance of additional investigation into the role of melatonin in liver health and its possible medicinal uses.

## 5 Melatonin and inflammation in the liver

**FIGURE 3:**
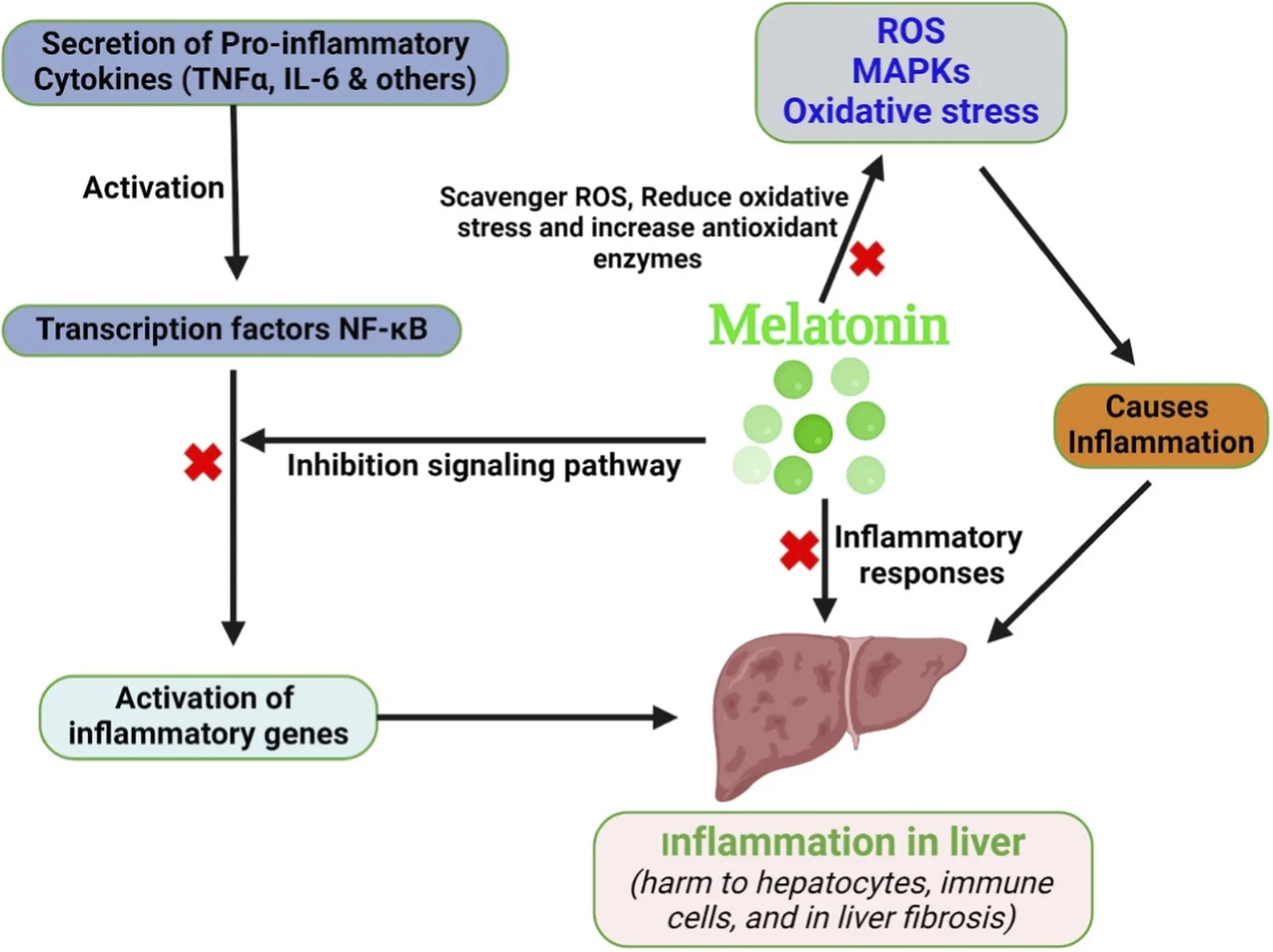
Mechanisms of melatonin for improving liver inflammation. Environmental stimuli and pro-inflammatory cytokines activate transcription factors (NF-kB, MAPKs, and others) and oxidative stress (ROS). The critical relationship between melatonin and inflammation in the liver is established because melatonin can inhibit different transcription factors and ROS is responsible for the activation of inflammatory genes.

Chronic inflammation plays a vital role in the advancement of several liver illnesses, such as hepatitis, steatohepatitis, and cirrhosis (Luci et al., 2020). The liver’s inflammatory processes are caused by the secretion of pro-inflammatory cytokines, including tumor necrosis factor-alpha (TNF-α), interleukin-6 (IL-6), and other agents. These cytokines activate signaling pathways such as nuclear factor kappa-light-chain-enhancer of activated B cells (Park et al., 2025). The persistent inflammatory reaction causes harm to hepatocytes, attracts immune cells, and results in liver fibrosis (Akkız et al., 2024). Melatonin, renowned for its powerful anti-inflammatory qualities, plays a crucial role in regulating these inflammatory processes in the liver (Hosseinzadeh et al., 2024; Woldańska-Okońska and Koszela, 2024). The primary mechanism by which melatonin exerts its anti-inflammatory effects is through the inhibition of the NF-κB signaling pathway. NF-κB is a transcription factor that controls the activation of many genes that promote inflammation. Melatonin inhibits the activation of NF-κB, decreasing the production of TNF-α, IL-6, and other inflammatory cytokines (Liu et al., 2024a). This leads to a reduction in the inflammatory response (Figure 3). This inhibitory action safeguards hepatocytes from damage caused by inflammation and maintains the functionality of the liver. Table 3 provides a concise overview of the impact of melatonin on liver inflammation, including it signaling pathways, experimental methods, concentration parameters, and effectiveness.

**TABLE 3:** The role of melatonin in hepatic inflammation.

Study model	Doses	Signaling mechanism	Efficacy	Ref
Non-alcoholic steatohepatitis (NASH) Animals	10–20 mg/kg (injected)	Inhibition of NF-κB, reduced TNF-α and IL-6, inhibition of MAPKs	Reduced liver inflammation, lowered pro-inflammatory cytokines, improved liver histology	(Saad et al., 2024)
Alcoholic liver disease (Animal Models)	10 mg/kg (injected)	Inhibition of NF-κB, inhibition of oxidative stress pathways	Decreased hepatic inflammation, lower cytokine levels, reduced oxidative damage	(Wang et al., 2024a)
Drug-Induced liver injury (Animal Models)	5–15 mg/kg (injected)	Inhibition of NF-κB and MAPKs, reduction of ROS	Reduced liver inflammation, lower oxidative stress, improved liver function	(Zhang et al., 2024b)
Chronic liver disease	3–10 mg/day	Inhibition of NF-κB, MAPK signaling, enhanced antioxidant enzymes	Decreased inflammation, hepatocyte protection, reduced liver fibrosis, and prevention of liver disease progression	(Mohammadian et al., 2024)
Hepatitis and Cirrhosis	10–20 mg/kg/day	Inhibition of NF-κB, regulation of SOD and GPx activity	Reduced cytokine levels, decreased oxidative stress, protection from hepatocyte damage	(Li et al., 2024)

Furthermore, melatonin impacts NF-κB and regulates various other signaling pathways that play a role in inflammation. It can inhibit the activation of mitogen-activated protein kinases (MAPKs), which generate inflammatory mediators (Abdelaal et al., 2024). Melatonin’capacity to block these pathways enhances its anti-inflammatory activity in the liver. The antioxidant properties of melatonin are also essential in its ability to reduce inflammation (Ain et al., 2024). Oxidative stress is a condition where there is an imbalance between ROS and antioxidant defenses (Singh et al., 2026). It plays a significant role in causing inflammation. Melatonin acts as a scavenger for ROS and boosts the effectiveness of antioxidant enzymes like SOD and GPx. Melatonin decreases oxidative stress, lowering oxidative damage and inflammatory signals that contribute to the pathogenesis of liver disease. Empirical research has shown that the injection of melatonin can effectively decrease hepatic inflammation in different animal models of liver disease (L et al., 2024a).

In NASH animals, melatonin administration decreased liver inflammation, lowered levels of pro-inflammatory cytokines, and enhanced liver histology (Dorranipour et al., 2024). Models of alcoholic liver disease and drug-induced liver injury have shown comparable anti-inflammatory effects of melatonin. However, the anti-inflammatory characteristics of melatonin indicate its potential as a medicinal agent for treating liver inflammation. Melatonin safeguards the liver from inflammation-induced harm by impeding crucial inflammatory pathways and diminishing oxidative stress. Consequently, it has the potential to hinder the advancement of chronic liver disorders. Additional clinical study is necessary to understand melatonin’s therapeutic capacity and enhance its utilization in treating liver disease.

## 6 Melatonin and liver fibrosis

Liver fibrosis is a crucial phase in the advancement of chronic liver diseases, characterized by the excessive buildup of extracellular matrix proteins, mainly collagen, which can result in cirrhosis and liver failure if not addressed (Addissouky et al., 2024; Kim et al., 2022; Bao et al., 2024). Liver fibrosis develops by activating hepatic stellate cells (HSCs), which transform into myofibroblast-like cells that generate collagen and other matrix components (Caon et al., 2024; Yum et al., 2023; Park et al., 2020). Oxidative damage, inflammation, and cytokine signaling promote this process. Melatonin, renowned for its robust antioxidant and anti-inflammatory characteristics, has emerged as a promising drug in the fight against liver fibrosis. Studies suggest that melatonin can alleviate the impact of oxidative stress in the liver by removing harmful free radicals and increasing the activity of antioxidant enzymes, including SOD and GPx.

**FIGURE 4:**
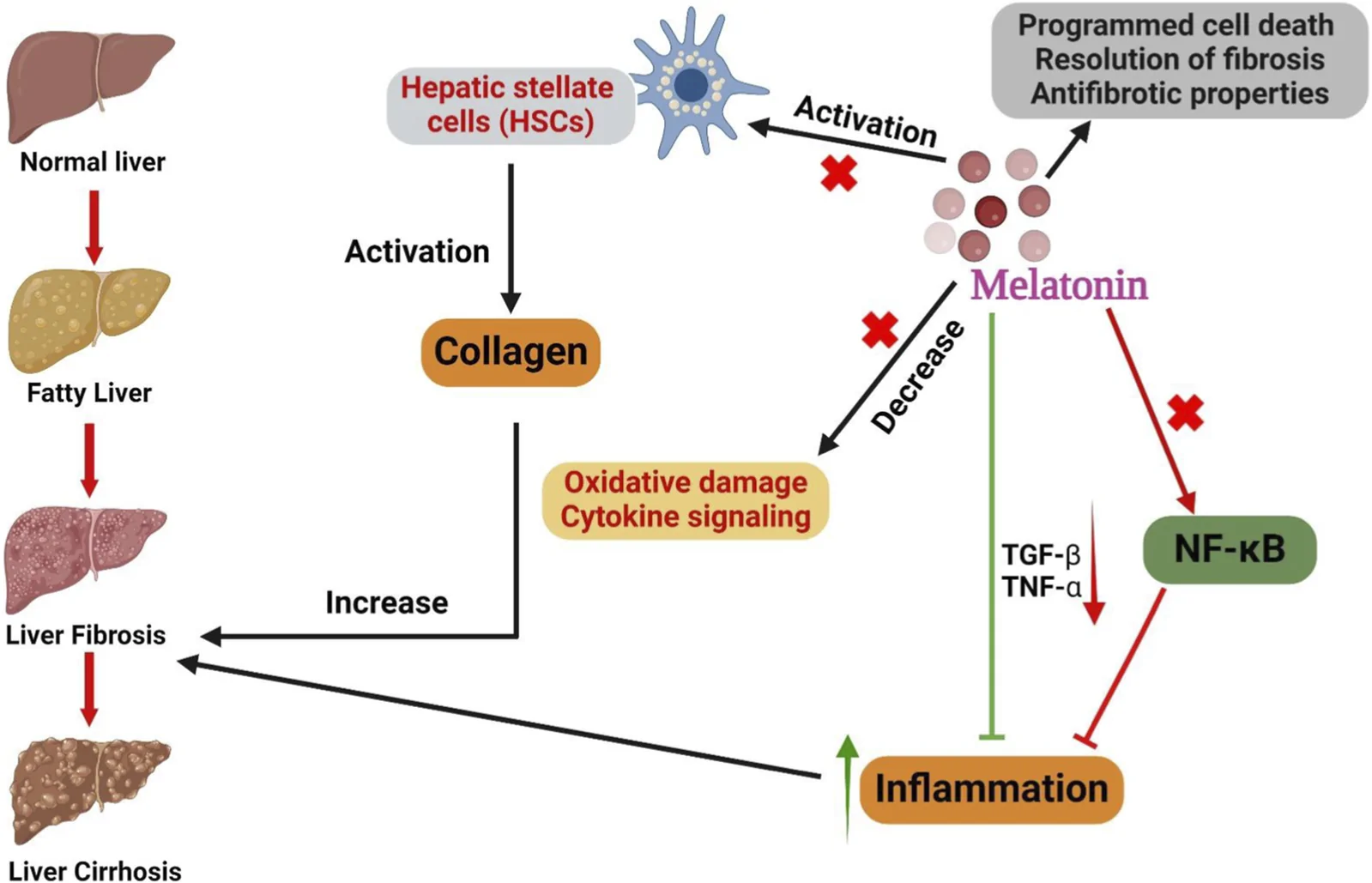
Melatonin fights against liver fibrosis by inactivating hepatic stellate cells and reducing oxidative stress and inflammation. Liver fibrosis is the post-stage of fatty liver and pre-stage of cirrhosis. Melatonin utilizes its antioxidant, anti-inflammatory, and direct anti-fibrotic properties to address liver fibrosis. It exerts antioxidant properties to decrease oxidative damage, inflammation, and cytokine signaling, stimulating HSCs and transforming HSCs into collagen and matrix components. On the other hand, melatonin reduces the inflammatory response associated with fibrosis progression by inhibiting the NF-kB pathway and TGF-β and TNF-α pathway. In addition, melatonin works against liver fibrosis by programmed cell death and fibrosis resolution.

Consequently, the decrease in oxidative stress can subsequently lead to a reduction in HSC activation. Melatonin has antioxidant properties and could reduce inflammation by influencing the production of pro-inflammatory cytokines, such as TGF-β and TNF-α, which play a crucial role in the development of fibrosis. By inhibiting the nuclear factor-kappa B (NF-κB) signaling pathway, melatonin effectively reduces the inflammatory response associated with fibrosis progression (Che et al., 2020), as NF-κB is a crucial regulator of inflammation (Figure 4). In addition, melatonin directly affects HSC behavior. Studies have demonstrated that it can hinder the growth and stimulation of HSCs, hence decreasing the production of collagen and other substances that make up the extracellular matrix. Melatonin additionally facilitates the programmed cell death of activated HSCs, hence assisting in the resolution of fibrosis (San‐Miguel et al., 2022). Studies conducted on animals with liver injury have shown strong evidence that melatonin has anti-fibrotic properties (Kim and Cheon, 2024). In models of carbon tetrachloride (CCl4)-induced liver fibrosis, melatonin administration effectively decreased fibrosis indicators and enhanced the histology of the liver (Rafiq et al., 2022). A comprehensive summary of the role of melatonin in liver fibrosis is shown in this Table 4, emphasizing melatonin’s effects on liver fibrosis from various research, including methods, models, concentrations, efficacy, animal model, duration, and molecular targets.

**TABLE 4:** Melatonin’s effects on liver fibrosis: processes, models, concentrations, and efficacy.

Process	Animal model	Doses	Duration	Efficacy	Molecular targets	Ref
Anti-fibrotic activity	Rat (carbon tetrachloride, CCl4) model	10 mg/kg/day (i.p.)	8 weeks	Reduced fibrosis, lower collagen deposition	TGF-β, α-SMA, Smad2/3, MMP-2, TIMP-1	(Liu et al., 2024b)
Antioxidant protection	Mouse (Bile duct ligation, BDL)	10 mg/kg/day (oral)	4 weeks	Decreased oxidative stress, reduced liver damage	Nrf2, HO-1, GSH, superoxide dismutase (SOD), catalase	(Li et al., 2023)
Inhibition of hepatic stellate cell activation	Rat (Thioacetamide-induced fibrosis)	20 mg/kg/day (oral)	6 weeks	Decreased stellate cell activation, reduced collagen buildup	Inhibition of TGF-β, downregulation of NF-κB, inhibition of ERK1/2 signaling	(Liu et al., 2024b)
Anti-inflammatory effects	Rat (CCl4-induced liver fibrosis)	10 mg/kg/day (i.p.)	10 weeks	Lowered levels of inflammatory cytokines	TNF-α, IL-6, COX-2, NF-κB	(Al-Rasheed et al., 2016)
Mitochondrial protection	Mouse (BDL)	5 mg/kg/day (i.p.)	2 weeks	Reduced mitochondrial damage, improved liver function	Mitochondrial ROS, Cytochrome c, Caspase-3	(Luo et al., 2019)
Inhibition of lipid peroxidation	Rat (CCl4 model)	10 mg/kg/day (oral)	8 weeks	Reduced malondialdehyde (MDA), enhanced antioxidant enzyme activity	Lipid peroxidation markers (MDA), SOD, catalase	(Li et al., 2023)
Fibrogenic cytokine suppression	Mouse (BDL model)	15 mg/kg/day (oral)	4 weeks	Reduced levels of fibrogenic cytokines	TGF-β1, TIMP-1, MMP-13	(San‐Miguel et al., 2022)
Autophagy modulation	Mouse (CCl4-induced fibrosis)	10 mg/kg/day (oral)	6 weeks	Enhanced autophagy, reduced fibrosis	mTOR inhibition, enhanced LC3-II, Beclin-1 activation	(Rahman et al., 2024)

Comparable findings have been noted in experiments with bile duct ligation (BDL) mice, where melatonin administration reduced fibrosis and reinstated normal liver function. Although there are encouising preclinical results, the available clinical data on the effectiveness of melatonin in treating liver fibrosis are currently scarce. Nevertheless, due to melatonin’s favorable safety profile and complex array of modes of action, it is an appealing option for further exploration in clinical trials. Therefore, melatonin provides a comprehensive strategy for addressing liver fibrosis by utilizing its antioxidant, anti-inflammatory, and direct anti-fibrotic properties (Piekarska et al., 2023). Further investigation and rigorous clinical assessment are necessary to fully exploit its potential in managing liver fibrosis and enhance patient outcomes.

## 7 Melatonin and liver cancer progression

**FIGURE 5:**
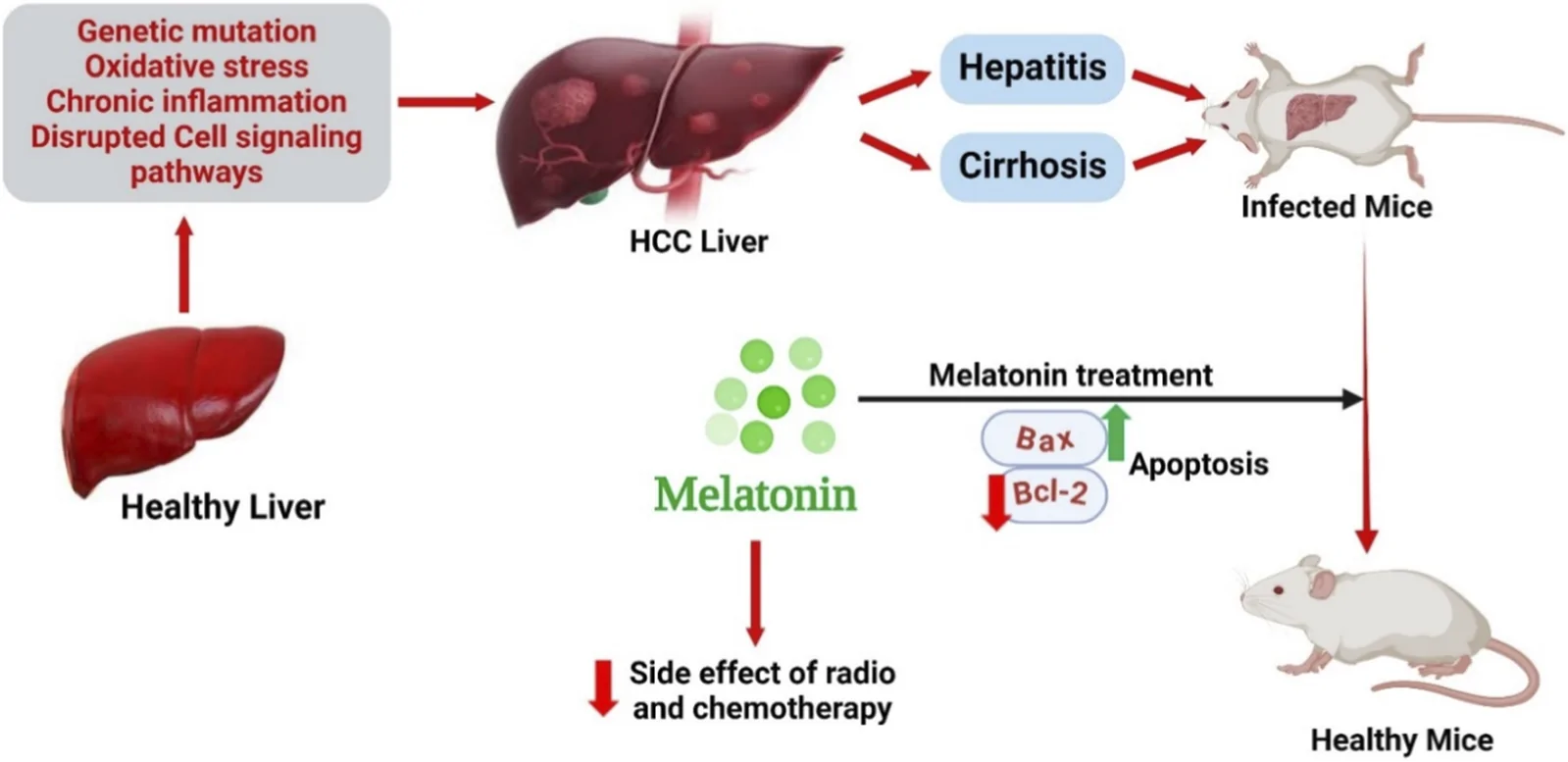
Critical relationship between the progression of liver cancer and melatonin. A model’s healthy liver experiences a physical or genetic abnormality that leads to hepatocellular carcinoma, which progresses to cirrhosis and hepatitis. A melatonin supplement causes apoptosis by upregulating and downregulating the apoptotic proteins Bax and Bcl-2. Melatonin exhibits various biological effects, including the inhibition or reduction of angiogenesis, cell proliferation, the cell cycle, VEGF (vascular endothelial growth factor), and tumor formation. Reduced tumor growth reduces metastasis. Additionally, melatonin lessens the effects of chemotherapy and radiation. Melatonin treatment may result in a healthy liver in a model of infected mice.

Hepatocellular carcinoma (HCC) is the prevailing form of liver cancer that frequently develops in individuals with chronic liver disorders, such as hepatitis and cirrhosis (Vaz et al., 2024; Mak et al., 2024; Phoolchund and Khakoo, 2024). HCC advances for multiple reasons, such as genetic mutations, oxidative stress, chronic inflammation, and disrupted cell signaling pathways (Choi et al., 2026). Melatonin, renowned for its high antioxidant capabilities, has attracted attention due to its potential role in impeding the advancement of liver cancer. Melatonin exerts its anti-cancer properties in multiple ways (Yi et al., 2024). One of the main functions of melatonin is to trigger apoptosis in cancer cells (Das et al., 2024). Apoptosis, also known as programmed cell death, is an essential mechanism that aids in removing damaged or malignant cells. Melatonin induces intrinsic apoptotic pathways by upregulating the expression of pro-apoptotic proteins such as Bax and downregulating the levels of anti-apoptotic proteins like Bcl-2 (L et al., 2024b; Saddam et al., 2024). This change in the equilibrium of apoptotic regulators facilitates the demise of HCC cells. Furthermore, melatonin triggers apoptosis and hinders cell proliferation, a crucial feature of cancer advancement. Melatonin disrupts the activity of multiple cell cycle regulators, including cyclins and cyclin-dependent kinases, resulting in the halting of the cell cycle at various stages. Melatonin inhibits the proliferation of HCC cells, hence decelerating tumor growth and progression (Figure 5).

Another significant anti-cancer action of melatonin is its ability to inhibit angiogenesis, forming new blood vessels that supply tumors with nutrients and oxygen (Goradel et al., 2017; Davoodvandi et al., 2024; Ma et al., 2020). Melatonin reduces the production of vascular endothelial growth factor (VEGF), which stimulates the formation of new blood vessels crucial for tumor growth and spread (Ma et al., 2020; Khafaga et al., 2024). Moreover, melatonin can potentially enhance the effectiveness of conventional cancer treatments, such as chemotherapy and radiotherapy, while reducing their adverse effects. It achieves this by protecting normal cells from oxidative damage caused by these treatments, thereby improving the overall therapeutic index. Preclinical studies have demonstrated that melatonin can impede tumor growth, reduce metastasis, and improve survival rates in animal models of HCC (Yi et al., 2024; Fernández-Palanca et al., 2021). These findings underscore the potential of melatonin as a beneficial addition to liver cancer treatment. However, it is essential to conduct clinical trials to confirm these benefits in patients and determine the most effective dosage methods and long-term safety. In summary, the diverse anti-cancer properties of melatonin position it as a highly promising option for both preventing and treating hepatocellular carcinoma. Its ability to induce programmed cell death, inhibit cell growth, prevent the formation of new blood vessels, and enhance the effectiveness of traditional treatments suggests that it could be a significant factor in the treatment of liver cancer. Additional research and clinical studies are crucial to fully exploit the potential of melatonin in preventing HCC. The following Table 5 summarizes the anticancer mechanisms of melatonin through antioxidant signaling in liver cancer growth, encompassing dosage, animal model, duration, and molecular targets.

**TABLE 5:** Antitumor mechanism of Melatonin via antioxidant signaling in liver cancer progression.

Doses	Animal model	Duration	Molecular targets	Antitumor mechanism via antioxidant signaling	Ref
10 mg/kg/day	Hepatocellular carcinoma rat model	6 weeks	NF-κB, Nrf2, SOD, GSH, Bax, Bcl-2	Melatonin increased Nrf2, boosting antioxidant defense (SOD, GSH) and decreased inflammation by downregulating NF-κB. It modulated Bax/Bcl-2 ratio to induce apoptosis	(de Almeida Chuffa et al., 2024)
20 mg/kg/day	DEN-induced liver cancer in rats	8 weeks	p53, caspase-3, MMP-9, Nrf2	Melatonin inhibited metastasis by activating p53 and caspase-3 to cause apoptosis and lower MMP-9 production. Nrf2 upregulation increased antioxidant response	(Liu et al., 2024b)
5 mg/kg/day	H22 hepatoma-bearing mice	4 weeks	ROS, SOD, catalase, GPx, ERK1/2	Melatonin lowered ROS, enhanced SOD and catalase activity, and inhibited ERK1/2 signaling, inhibiting tumor growth and oxidative stress	(Wang et al., 2024b)
10 mg/kg/day	HepG2 xenograft mouse model	6 weeks	AMPK, Nrf2, HO-1, NF-κB	Melatonin reduces inflammation and oxidative damage by activating AMPK, upregulating Nrf2/HO-1 antioxidant pathway, and suppressing NF-κB, ultimately preventing tumor progression	(Zhao et al., 2024)
50 mg/kg/day	DENA-induced hepatocarcinogenesis in rats	10 weeks	Nrf2, COX-2, iNOS, TNF-α	Melatonin reduced oxidative stress, inflammation, and tumor promotion by increasing Nrf2 activity and downregulating COX-2, iNOS, and TNF-α	(Pan et al., 2024)

## 8 Melatonin-based combination therapies in liver diseases

The combined therapy of melatonin in disorders of the liver demonstrates potential in both animal models and clinical trials, mainly due to its antioxidant, anti-inflammatory, and anti-fibrotic effects. The diverse features of melatonin, such as its ability to act as an antioxidant, reduce inflammation, and prevent fibrosis, make it an up-and-coming option for treating several liver disorders (Millet-Boureima et al., 2021). Melatonin has demonstrated considerable potential in the treatment of NAFLD (Terziev and Terzieva, 2023; Rezayat et al., 2021). Empirical research has shown that adding melatonin can decrease the amount of fat in the liver, enhance the levels of liver enzymes, and improve the histological characteristics of NASH (Martínez Soriano et al., 2020; Miguel et al., 2022; Wang et al., 2024c). The hormone’s capacity to enhance mitochondrial function and decrease oxidative stress is pivotal in producing these advantageous effects. Studies have also examined the involvement of melatonin in ALD, and prolonged alcohol intake results in oxidative stress, inflammation, and consequent liver damage (de Almeida Chuffa et al., 2024; Michalak et al., 2021). Studies conducted on individuals with ALD have demonstrated that melatonin can enhance liver function tests and decrease indicators of oxidative damage. In addition, melatonin’s anti-inflammatory qualities aid in reducing alcohol-induced inflammation in the liver, thereby offering protection against the advancement of liver disease (Ozturk et al., 2023; Vohra et al., 2021). Numerous clinical trials and animal research have investigated its potential, especially regarding its capacity to regulate oxidative stress, inflammation, and apoptosis, all of which are essential in liver pathology.

Melatonin has demonstrated potential in laboratory animal models for liver fibrosis and cirrhosis, while limited clinical data is available (Terziev and Terzieva, 2023; Ferreira et al., 2021). Studies conducted on animals have shown that melatonin can limit the production of fibrogenic cytokines and prevent the activation of hepatic stellate cells, reducing fibrosis (San‐Miguel et al., 2022; Zhang et al., 2023; Sohrabi et al., 2021). Further clinical trials are necessary to validate the effectiveness of melatonin in treating advanced liver fibrosis and cirrhosis in human patients despite the encouraging nature of these findings. The examination of melatonin’s role in HCC, which is the most prevalent form of primary liver cancer, is now underway (Mihanfar et al., 2022; Wang et al., 2024d). Preclinical investigations have demonstrated that melatonin could impede the growth of cancer cells, trigger programmed cell death, and augment the effectiveness of standard chemotherapy (Adedokun et al., 2024; An et al., 2024). Preliminary clinical trials indicate that the use of melatonin, along with other therapeutic drugs, may enhance overall survival and mitigate treatment-related side effects in patients with HCC (Hin Tang et al., 2020; Hassan et al., 2024).

Melatonin has also demonstrated synergistic effects when combined with metabolic interventions for MASLD and metabolic dysfunction-associated steatohepatitis (MASH). Clinical studies indicate that melatonin supplementation together with dietary modification and physical exercise produces greater improvements in serum ALT, AST, insulin resistance, lipid metabolism, and hepatic steatosis than lifestyle intervention alone (Bahrami et al., 2020; Charatcharoenwitthaya et al., 2021). These benefits are mediated through activation of the Nrf2/HO-1 antioxidant pathway, suppression of NF-κB-dependent inflammation, activation of AMPK, inhibition of SREBP-1c-mediated lipogenesis, and enhancement of mitochondrial β-oxidation (Fernández et al., 2015). In experimental liver fibrosis, melatonin enhances the efficacy of established anti-fibrotic strategies by suppressing TGF-β/Smad signaling, reducing hepatic stellate cell activation, decreasing α-SMA expression, and limiting extracellular matrix deposition (Hosseinzadeh et al., 2024). Combination treatment with antioxidants or hepatoprotective compounds further attenuates oxidative injury and inflammatory responses, suggesting complementary mechanisms of action that may delay fibrosis progression (San-Miguel et al., 2022).

In HCC, melatonin has emerged as a promising chemosensitizing and radiosensitizing agent. Numerous preclinical studies have demonstrated that melatonin potentiates the antitumor activity of sorafenib, doxorubicin, cisplatin, and 5-fluorouracil by enhancing apoptosis, promoting cell-cycle arrest, suppressing angiogenesis, and reducing multidrug resistance (Lin et al., 2017; Fernandez-Palanca et al., 2021). Melatonin also improves mitochondrial function, increases ROS-mediated cancer cell death, inhibits epithelial-mesenchymal transition (EMT), and enhances immune surveillance through modulation of inflammatory signaling pathways (Nuszkiewicz et al., 2026). Furthermore, melatonin may reduce treatment-associated toxicity, thereby improving the therapeutic index of conventional anticancer therapies (Martínez Ruiz, 2024).

Beyond pharmacological combinations, melatonin has shown potential when integrated with nutraceuticals and natural antioxidants, including vitamin E, resveratrol, curcumin, and omega-3 fatty acids (Dadwal et al., 2025). These combinations synergistically strengthen endogenous antioxidant defenses by increasing SOD, GPx, and catalase (CAT) activity while reducing lipid peroxidation and mitochondrial dysfunction. Although most evidence remains preclinical, these findings highlight the potential of multi-target therapeutic strategies.

### 8.1 Preclinical evidence: mechanistic and animal studies

Comprehensive preclinical studies have revealed the liver-protective capabilities of melatonin in various experimental models of liver disease, such as alcoholic liver disease (ALD), metabolic dysfunction-associated steatotic liver disease (MASLD/NAFLD), drug-induced liver damage, hepatic fibrosis, cirrhosis, and hepatocellular carcinoma (Che et al., 2023). *In vitro* investigations utilizing hepatocytes, hepatic stellate cells, Kupffer cells, and liver cancer cell lines have reliably demonstrated that melatonin mitigates oxidative stress by directly neutralizing reactive oxygen and nitrogen species while stimulating intrinsic antioxidant defense mechanisms via the Nrf2/HO-1 pathway (Sato et al., 2020). Melatonin additionally diminishes inflammatory signaling by obstructing NF-κB activation and lowering the synthesis of pro-inflammatory cytokines such as TNF-α, IL-1β, and IL-6 (Nabavi et al., 2019). In animal studies, melatonin enhances hepatic steatosis, reduces lipid peroxidation, revitalizes mitochondrial function, suppresses hepatic stellate cell activation, and diminishes extracellular matrix accumulation via modulation of the TGF-β/Smad signaling pathway (Hu et al., 2019). The advantageous outcomes are usually attained through the administration of comparatively elevated dosages of melatonin, commonly from 5 to 50 mg/kg/day, contingent upon the species, disease model, method of delivery, and length of treatment (Ahmad et al., 2023). The necessity for elevated dosages in experimental animals mostly stems from species-specific pharmacokinetics, swift metabolic elimination, and the acute clinical states elicited in laboratory models (Srirangan and Sabina, 2025). These studies collectively demonstrate that melatonin protects the liver through multiple complementary mechanisms targeting oxidative stress, inflammation, mitochondrial dysfunction, metabolic dysregulation, fibrosis, and tumor progression. Together, these investigations furnish compelling mechanistic proof endorsing melatonin as a viable treatment option for the prevention and management of liver ailments.

Preclinical investigations provide the biological foundation supporting the therapeutic potential of melatonin in liver diseases. These mechanistic discoveries have been reliably corroborated in animal models of alcoholic liver disease, MASLD/MASH, drug-induced liver damage, fibrosis, cirrhosis, and hepatocellular cancer. The administration of melatonin, often between 5 and 50 mg/kg/day, markedly diminishes hepatic steatosis, oxidative stress, inflammatory cell infiltration, collagen accumulation, and tumor load, while enhancing liver histology and biochemical indicators of hepatic damage (Terziev and Terzieva, 2023). These protective benefits are associated with heightened activity of intrinsic antioxidant enzymes, such as superoxide dismutase (SOD), catalase (CAT), glutathione peroxidase (GPx), and heme oxygenase-1 (HO-1) (Mathes, 2010). Moreover, numerous studies have shown that the combination of melatonin with standard medications produces synergistic benefits, leading to greater antioxidant protection, decreased fibrosis, and increased treatment effectiveness (Cinar et al., 2025). Together, these strong mechanistic and *in vivo* results offer persuasive proof endorsing the clinical application of melatonin for the prevention and management of chronic liver ailments.

### 8.2 Clinical evidence and translational perspectives

Clinical research examining melatonin in individuals with hepatic disorders is still somewhat scarce, although has yielded promising results. Randomized controlled trials and preliminary clinical investigations have assessed oral melatonin supplementation, generally at doses between 2 and 10 mg per day, in individuals with MASLD/NAFLD, cirrhosis, viral hepatitis, and various chronic liver ailments (Al-Mosawi et al., 2026). These investigations have indicated enhancements in blood liver enzymes, such as alanine aminotransferase (ALT) and aspartate aminotransferase (AST), alongside decreases in oxidative stress indicators, inflammatory agents, and insulin resistance. Melatonin has shown beneficial impacts on sleep quality, circadian rhythm regulation, and general quality of life in individuals with chronic liver disease (Sato et al., 2020). Significantly, melatonin demonstrates a commendable safety record, presenting minimal side effects even with extended use. Nonetheless, the existing clinical data is limited by modest sample numbers, diverse trial cohorts, inconsistent treatment durations, and variations in dosage protocols (Tuft et al., 2023). Moreover, the diminished dosages administered to people in contrast to animal research illustrate variances in pharmacokinetics, metabolic rates, and recognized clinical safety parameters, rendering direct dose comparisons unsuitable without allometric scaling. Consequently, extensive multicenter randomized controlled trials are necessary to determine the ideal therapeutic dosage, treatment length, long-term safety, and effectiveness of melatonin, both as a standalone treatment and in conjunction with current pharmaceutical therapies for chronic liver conditions (De Silva et al., 2020). Synopsis of clinical research analyzing melatonin in liver conditions and corresponding metabolic disorders are presented in Table 6.

**TABLE 6:** Summary of clinical studies evaluating melatonin in liver diseases and related metabolic disorders.

Disease/Patient cohort	Study design/Phase	Sample size (n)	Melatonin treatment	Co-treatment	Primary clinical outcomes	Main findings	Clinical Trials.gov Id	Ref
Non-alcoholic fatty liver disease (NAFLD/MASLD)	Randomized controlled trial	74	10 mg/day orally for 4 months	Standard care	ALT, AST, GGT, lipid profile	Significant improvement in liver enzymes and oxidative stress markers	Not reported	(Celinski et al., 2014)
Non-alcoholic steatohepatitis (NASH/MASH)	Prospective randomized study	97	5 mg twice daily for 14 months	Lifestyle modification	Liver enzymes, inflammatory markers	Reduced aminotransferases and improved inflammatory status	Not reported	(Celinski et al., 2014)
NAFLD with metabolic syndrome	Double-blind placebo-controlled trial	100	6 mg/day for 12 weeks	Diet and exercise	ALT, AST, insulin resistance (HOMA-IR), hs-CRP	Improved liver function, insulin sensitivity and inflammation	Not reported	(Bahrami et al., 2020)
Liver cirrhosis with sleep disturbance	Clinical study	40	5 mg/day for 8 weeks	Conventional therapy	Sleep quality, circadian rhythm	Improved sleep quality and reduced fatigue	Not reported	(De Silva et al., 2020)
Chronic hepatitis C	Randomized clinical trial	60	Melatonin 3 mg/day	Standard antiviral therapy	Viral response, ALT, AST	Enhanced antioxidant status and improved liver enzymes	Not reported	(El-Mahdy et al., 2023)
Alcoholic liver disease	Pilot clinical studies	20–60	3–10 mg/day	Conventional treatment	Oxidative stress biomarkers, liver enzymes	Improved antioxidant capacity; limited evidence due to small sample size	Not reported	(Bahrami et al., 2020)
MASLD and chronic liver disease	Early clinical evaluation	Various	2–10 mg/day	Combination therapy	Safety, liver function, oxidative stress	Results pending or unavailable	Include NCT identifiers where available	(Esmaeili et al., 2021)

The promising findings obtained from cellular and animal studies have provided the rationale for evaluating melatonin in human clinical trials. Future investigations should focus on large multicenter randomized controlled trials employing standardized treatment protocols and clearly defined clinical endpoints. Particular attention should be directed toward identifying predictive biomarkers of therapeutic response, optimizing dosage and treatment duration, evaluating long-term safety, and determining the efficacy of melatonin as an adjunctive therapy in combination with established pharmacological interventions. Establishing this translational continuum from mechanistic evidence to clinical application will facilitate the development of evidence-based melatonin therapies for chronic liver diseases.

## 9 Future perspectives of melatonin research and clinical application in liver diseases

The potential of melatonin as a therapeutic agent in liver diseases presents numerous attractive opportunities for future research and practical implementation. As our comprehension of melatonin’s molecular mechanisms improves, numerous crucial domains require further investigation to use its therapeutic capacity properly. Firstly, it is necessary to conduct thorough studies to clarify the exact signaling pathways and molecular targets by which melatonin influences liver cells. Studying these pathways can offer a valuable understanding of how melatonin influences oxidative stress, inflammation, and fibrosis on a cellular level. Acquiring this knowledge is essential for developing precise treatments that can optimize the advantages of melatonin in treating liver diseases. Furthermore, investigating the combined effects of melatonin with current treatments for liver disease can improve therapeutic results (Fayazi et al., 2024; Abdallah et al., 2024). Co-administering Melatonin with conventional therapies, such as anti-inflammatory medications, antifibrotic substances, or chemotherapy drugs, can enhance effectiveness and minimize adverse reactions. Conducting clinical studies to evaluate these combination medicines is crucial for identifying the most efficient treatment procedures and establishing consistent dose regimes (Fourie Zirkelbach et al., 2022).

Research on developing new melatonin analogs and delivery mechanisms is also crucial. Improving the ability of melatonin to be absorbed and remain stable in the body can significantly enhance its effectiveness as a treatment (Talib et al., 2021; Biggio et al., 2021). This can be achieved by creating analogs or using modern delivery methods like nanoparticles or liposomes (Reinsalu et al., 2024). These advancements have the potential to enable more accurate localization of liver tissues, hence decreasing overall exposure and mitigating any adverse reactions. Furthermore, it is imperative to consider personalized medicine approaches when researching melatonin (Klerman et al., 2020). Optimizing treatment outcomes can be achieved by identifying specific patient populations who would derive the most significant benefit from melatonin therapy, considering genetic, metabolic, or disease-specific characteristics. Utilizing biomarker studies and pharmacogenomic research will be crucial in attaining this objective (Al et al., 2023; Fekry et al., 2024). Finally, it is imperative to conduct extensive and prolonged clinical trials to validate melatonin’s safety and effectiveness in treating different liver conditions. These studies should focus on establishing evidence-based recommendations for using melatonin in clinical practice, considering factors such as the most effective dosage, duration of treatment, and possible interactions with other drugs. To summarize, future studies on melatonin in liver diseases should prioritize understanding its molecular underpinnings, investigating combination treatments, creating innovative delivery systems, and implementing personalized medicine strategies. By implementing these initiatives, melatonin can become a fundamental element in treating liver diseases, promising enhanced patient results and quality of life.

## 10 Conclusion

Melatonin, a hormone synthesized mainly by the pineal gland, demonstrates significant chemopreventive capabilities in several liver disorders due to its high antioxidant effects. The compound’s capacity to collect free radicals, boost the activities of antioxidant enzymes, and regulate inflammatory responses makes it a potent agent in combating liver damage caused by oxidative stress. Melatonin attenuates oxidative stress and inflammation in alcohol-induced liver disorders, thereby preserving hepatic function. Additionally, it benefits metabolic pathways, enhancing insulin sensitivity and lipid metabolism, which play a crucial role in managing NAFLD. The anti-inflammatory properties of melatonin are achieved by suppressing pro-inflammatory cytokines and signaling pathways. This protection helps prevent chronic liver inflammation, a significant factor in advancing liver disease. The therapeutic potential of this substance is emphasized by its ability to reduce the activation of hepatic stellate cells and the production of fibrogenic cytokines due to its antifibrotic capabilities. In addition, melatonin exhibits substantial anti-cancer properties, making it a promising supplement in treating hepatocellular carcinoma. This is due to its ability to induce programmed cell death, inhibit cell growth, and prevent the formation of new blood vessels. However, initial clinical trials have commenced to substantiate the effectiveness and safety of melatonin in managing liver disease, although further comprehensive research is necessary. Subsequent investigations should prioritize the identification of specific molecular targets, the refinement of dosing schedules, and the creation of sophisticated delivery mechanisms. Therefore, incorporating melatonin into therapeutic approaches for liver diseases has the potential to significantly enhance patient outcomes, representing a notable progression in managing liver diseases.
